# A Case Report of Severe Osteomalacia in a Young Patient After Bariatric Surgery

**DOI:** 10.7759/cureus.20198

**Published:** 2021-12-06

**Authors:** Hamzah M Alarfaj, Wedyan Y Alrasheed, Sumaiyah A Alsulaiman, Fai T Almulhem, Meriam F Almaideni, Khalid W Alkuwaity

**Affiliations:** 1 Internal Medicine, King Fahad Hospital - Al-Hofuf, Al-Ahsa, SAU; 2 Medicine, King Faisal University, Al-Ahsa, SAU; 3 Orthopedics, King Fahad Hospital - Al-Hofuf, Al-Ahsa, SAU

**Keywords:** metabolic bone disease, fracture, hyperparathyroidism, bone mineral density (bmd), vitamin d, calcium, biliopancreatic diversion, obesity

## Abstract

Obesity can promote several metabolic, cardiovascular, and musculoskeletal complications and has been associated with poor quality of life. The treatment of obesity can range from simple lifestyle modifications or medications to complicated bariatric surgeries. Although bariatric surgery has been a proven treatment for morbid obesity, it has also been associated with multiple consequences and complications. Several reports and studies have revealed bone loss or decreased bone mineral density (BMD), fractures, or even several metabolic bone diseases, such as osteoporosis, following bariatric surgery. This case report aims to increase awareness on postoperative patient supplementation compliance and incorporation of early detection and intervention. This case report involves a 39-year-old male who underwent laparoscopic biliopancreatic diversion 10 years prior to presentation. The patient was not compliant with his supplements for over nine years, which lead to multiple fragility fractures, myopathy, and muscle atrophy due to hypocalcemia, vitamin D deficiency, hyperparathyroidism, and other electrolyte disturbances. He has since been treated with supplements and physiotherapy for 10 months and showed clinical improvement. This case report highlights the importance of pre- and postoperative screening of bone loss risks and any vitamin or mineral deficiencies with subsequent correction via supplements. Moreover, it emphasizes the need for more studies on the complications of late post-bariatric surgeries.

## Introduction

Obesity is a multifactorial disease that exerts a powerful influence on all functions of the human body. Moreover, it raises the risk for various multisystemic medical conditions, including cardiovascular disease, certain cancers, musculoskeletal disorders, metabolic disease, and poor mental health, which eventually lead to lower quality of life. Globally, obesity is not an uncommon concern. Indeed, one study showed an obesity prevalence of 40% in adults between 20 and 39 years and over 44% in adults aged between 40 and 59 years [1]. Local estimates have shown that more than 24% of the Saudi population would be obese in 2019 [2]. Obesity can be managed by lifestyle modification, medication, and surgery. Although the first two promote less risk, they have limited effectiveness and more frequently cause relapse. In contrast, bariatric surgeries are more effective for weight loss and have been associated with a significant reduction in comorbidities [3]. The International Federation for the Surgery of Obesity and Metabolic Disorders (IFSO) showed that more than 833,000 bariatric surgeries had been performed worldwide in 2019 [4]. According to the Saudi Journal of Obesity, approximately 15,000 bariatric surgeries are performed every year in Saudi Arabia [5,6]. Several bariatric surgeries are available. The most frequently performed bariatric surgeries worldwide are sleeve gastrectomy (SG) and Roux-en-Y gastric bypass (RYGB) [4]. Sleeve gastrectomy (SG) involves subtotal vertical gastrectomy with conservation of the pylorus, including longitudinal removal of the fundus, corpus, and antrum, to create a cylindrical duct along the lesser curvature [7]. On the other hand, the gastric bypass procedure, also known as RYGB, involves creating a small proximal gastric pouch by dissecting the distal portion of the stomach and then anastomosing the gastric pouch with the jejunum [8]. The beneficial outcomes of losing weight include the significant reduction in the risk of comorbidities, including cardiovascular disease, hypertension, obstructive sleep apnea, and inflammation. Furthermore, some bariatric surgery has been proven to improve glucose profile to prediabetic level for type 2 diabetes mellitus [9]. Despite its ability to reduce significant comorbidities, bariatric surgery can nevertheless lead to numerous postoperative complications [3]. Multiple factors can increase the risk of morbidities after surgeries, including age, male sex, comorbid chronic diseases, very high body mass index (BMI), surgeon experience, quality and experience of the center and facility, and open surgical approach. These complications can present early or later after surgery [10]. Early complications after surgery include bleeding, wound infections, anastomotic fistulas, and venous thromboembolism, such as deep vein thrombosis and pulmonary embolism. The mortality rate in the first 30 days after the surgery has generally remained <1%. The most common cause of death includes pulmonary embolism and surgical leakage [10]. Peritonitis due to an anastomotic fistula has been considered the most common complication [11]. Nutritional deficiencies, dumping syndrome, incisional and internal hernia, anastomotic stenosis, small bowel syndrome, small bowel obstruction, marginal ulcer, gastric erosions, cholelithiasis, and postoperative weight regain have all been considered as late complications after surgery [10,11]. Nutritional complications are more common in malabsorptive bariatric surgeries than in restrictive surgeries [11]. Moreover, nutritional complications can be caused either by reduced intake or malabsorption [12]. Examples of nutritional deficiencies include electrolyte disturbances (i.e., low calcium, magnesium, potassium, sodium, and phosphorus levels), deficiencies in fat-soluble vitamins (i.e., vitamins A, D, E, and K), and deficiencies in minerals such as folic acid and iron. Nutritional complications have various presentations, such as arrhythmias, myopathies, hypoglycemia, anemia, neuropathies, oxalosis and renal stones, secondary hyperparathyroidism, and metabolic bone disease [10]. The main factors for accelerated bone loss after bariatric surgery are calcium and vitamin D deficiencies with elevated parathyroid hormone [3,12]. The incidence rate of calcium deficiency after bariatric surgery is almost 10%, whereas the prevalence of vitamin D deficiency after bariatric surgery lies between 25% and 73% [12]. Researchers have suggested that bariatric surgery can exacerbate or induce vitamin D deficiency. The factors that can contribute to this complication include malabsorption, inadequate supplementation, nonadherence, reduced dairy product intake, vomiting, and excess adiposity [12,13]. Nevertheless, the prevalence of preoperative vitamin D deficiency has also remained high, ranging from 25% to 80% [12]. A previous study reported a significant improvement in postoperative vitamin D levels over four years of follow-up, which could have been due to the high level of compliance and rapid release of the sequestrated vitamin D in adipose tissue following weight loss. Other studies have shown that some patients remain deficient despite appropriate vitamin D supplementation and follow-up [13]. According to the American Association of Clinical Endocrinologist’s recommendation for supplements of calcium and vitamin D after bariatric surgery, patients whose serum 25-hydroxyvitamin D (25(OH)D) levels decrease below 30 ng/mL should be administered vitamin D supplements in the form of vitamin D2 or D3 in oral doses of 50,000 IU one to three times/week until sufficient levels are reached. This should be followed by additional maintenance supplementation of 2000-10,000 IU/day to attain serum levels of >30 ng/mL. They also recommend oral calcium supplementation with natural calcium in food to reach a total of 1500-20,000 mg/day [13]. Most suggest preoperative assessment and postoperative regular follow-up for supplemental dose adjustment and lifelong monitoring [10,11]. Although bariatric surgery has been considered a great option for losing weight, other proven treatments for obesity have been available besides lifestyle modifications. New studies have highlighted pharmacological options as a reliable treatment for obesity in adjunct to lifestyle modifications [14]. Orlistat, phentermine/topiramate extended-release capsule, and lorcaserin are long-term medical treatments for obesity approved by the Food and Drug Administration (FDA). Other FDA-approved options include diethylpropion, phendimetrazine, and benzphetamine [15]. Moreover, liraglutide, which is a glucagon-like peptide 1 (GLP-1) analog, has been approved by the FDA and European Medicines Agency as a medical treatment that promotes weight loss [14]. Age, sex, medical comorbidities, current medications, treatment response, side effect profile, drug efficacy, availability of long-term safety data, and cost have been identified as factors that influence the selection of medication in each patient [15]. This case report aims to increase awareness on postoperative patient supplementation compliance and incorporation of early detection and intervention. The rationale of this case is to avoid disastrous consequences on the patient’s health due to hypovitaminosis after bariatric surgery.

## Case presentation

A 39-year-old bedridden male presented to the emergency room with convulsions, severe shoulder and pelvic pain, and severe muscle weakness. He was admitted for seizures possibly caused by electrolyte imbalance. The medical history of the patient revealed that he was morbidly obese with a BMI of 53.1, for which he was treated surgically via laparoscopic biliopancreatic diversion 10 years prior to presentation. Postoperatively, the patient was well, stable, and kept on thiamine, calcium, and vitamin D supplements for one year. However, the patient stopped taking his supplements due to personal issues. Approximately two years after the surgery, he complained of fatigue and proximal muscle weakness. At three years after the surgery, he started complaining of numbness on his extremities, leg tremors with hand spasms, and more progressive upper and lower muscle weaknesses, for which he started using walking aids. Around that time, he sought medical advice and visited a general practitioner who provided reassurance and muscle relaxants without further evaluation or correction of electrolyte and vitamin disturbances. Consequently, his condition worsened with time until such a point that he became handicapped and completely bedridden five years after the surgery. Furthermore, the patient’s history indicated that he complained of confusion, memory loss, daytime somnolence, headaches, and disturbed sleep. Moreover, he complained of dysphagia with solid food, frequent constipation, insomnia, urinary incontinence, and history of bedsores in the right sciatic area, which was treated conservatively. On admission, the patient was alert and conscious but confused. The patient’s vital signs were stable with reduced oxygen saturation at 92% in room air. Upon general examination, the patient looked ill and in pain, with loose hair, stomatitis, glossitis, and general skin dryness. Musculoskeletal examination revealed general muscle wasting and left knee and right ankle deformities. Neurological examination revealed a 1/5 and 3/5 in lower and upper extremity muscle strength, respectively, with intact sensory function. Further systematic examinations were unremarkable. Initially, the patient was admitted to rule out neurological causes and electrolyte disturbances. Upon initial laboratory evaluation, severe vitamin D deficiency with severe hypocalcemia, secondary hyperparathyroidism, and different electrolyte disturbance was discovered as shown in Table 1.

**Table 1:** Laboratory test results before and after treatment.

	Before treatment	After treatment	Normal range
White blood cells (WBCs) (× 10^9^/L)	11.83	–	4–11
Mean corpuscular volume (MCV) (FL)	59.6	–	80–100
Hemoglobin (g/dL)	11.9	–	13–17
Calcium, total (mmol/L)	0.92	1.89	2.1–2.6
Sodium (mmol/L)	128	139	135–145
Potassium (mmol/L)	2.90	4.51	3.5–5
Chloride (mmol/L)	92	113.60	95–105
Vitamin B 12 (pg/mL)	285	714	180–914
Vitamin D3 (ng/mL)	0.1	29.2	30–100
Parathyroid hormone (pmol/L)	59.5	5.86	1.6–7
Magnesium (mmol/L)	0.24	0.85	0.85–1.10
Phosphorus (mmol/L)	0.56	1.09	0.81–1.58
Serum folate (ng/mL)	3.36	10.89	3–18
Alkaline phosphatase (ALP) (IU/L)	1017	217	50–136

**Figure 1:**
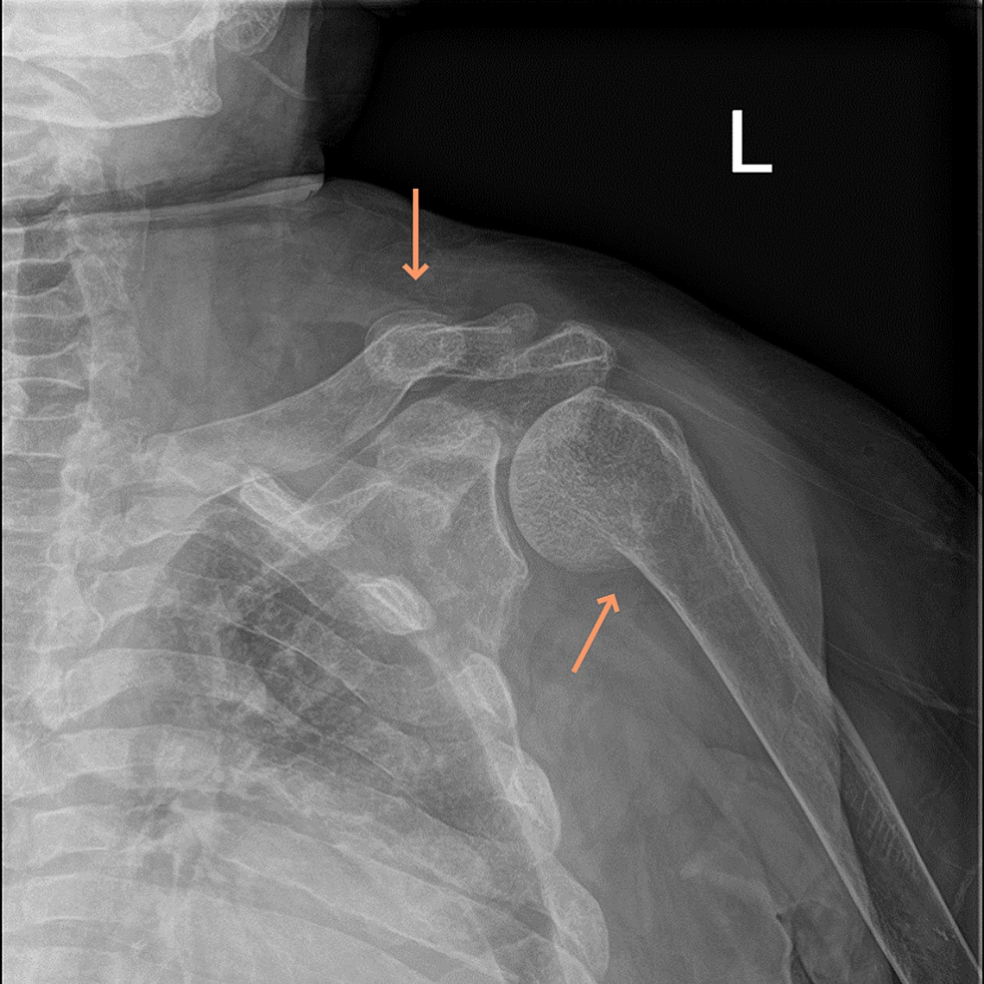
Left shoulder radiograph showing reduced bone density with a healed left clavicular fracture and a healed proximal humerus fracture (arrows).

**Figure 2:**
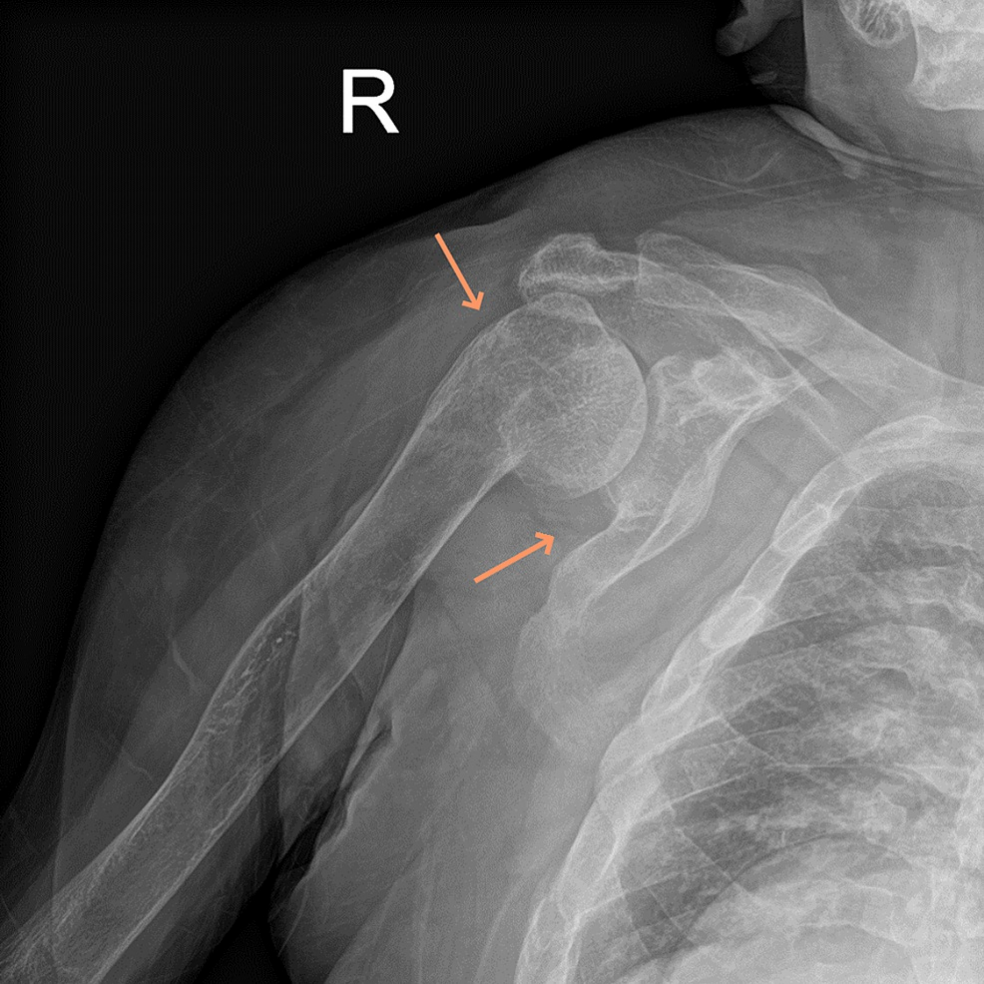
Right shoulder radiograph showing a healed right proximal humerus fracture in the varus position with a deformed scapular neck consistent with an old fracture (arrows).

**Figure 3:**
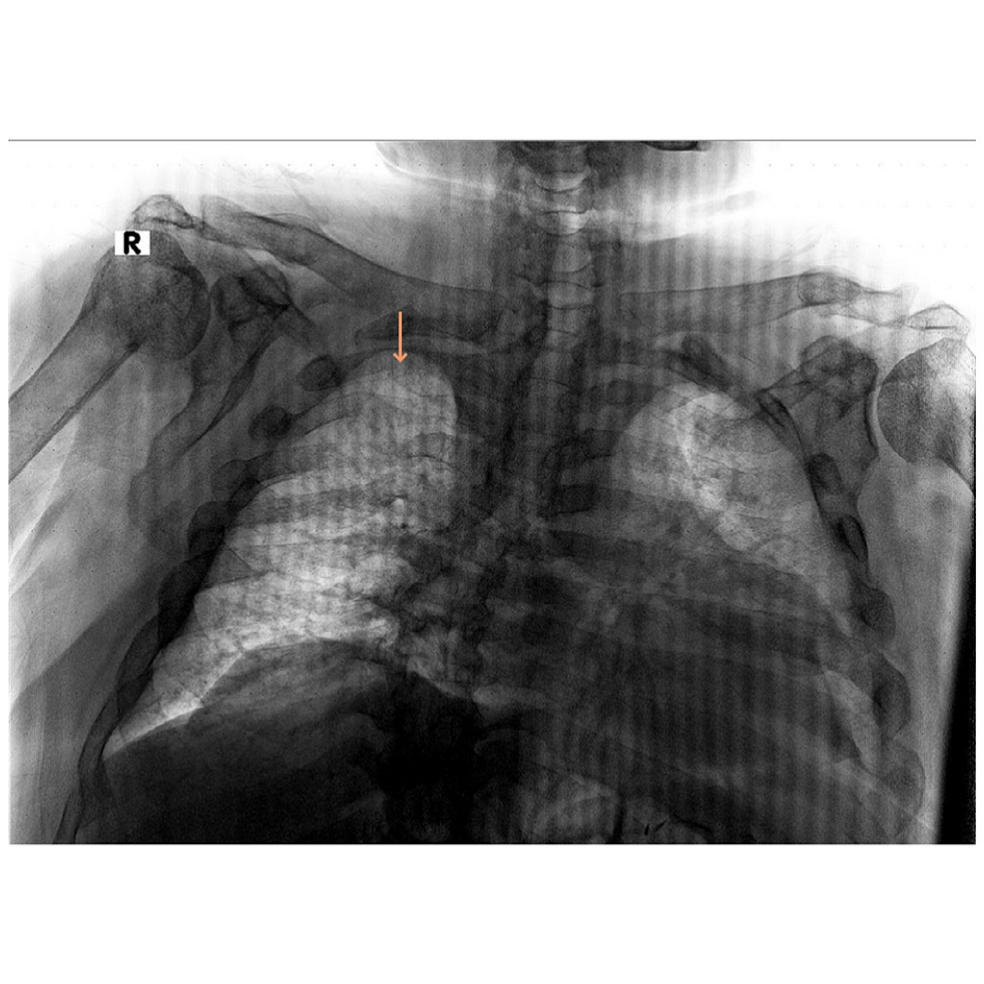
Chest radiograph showing healed right-sided multiple rib fractures (arrow).

**Figure 4:**
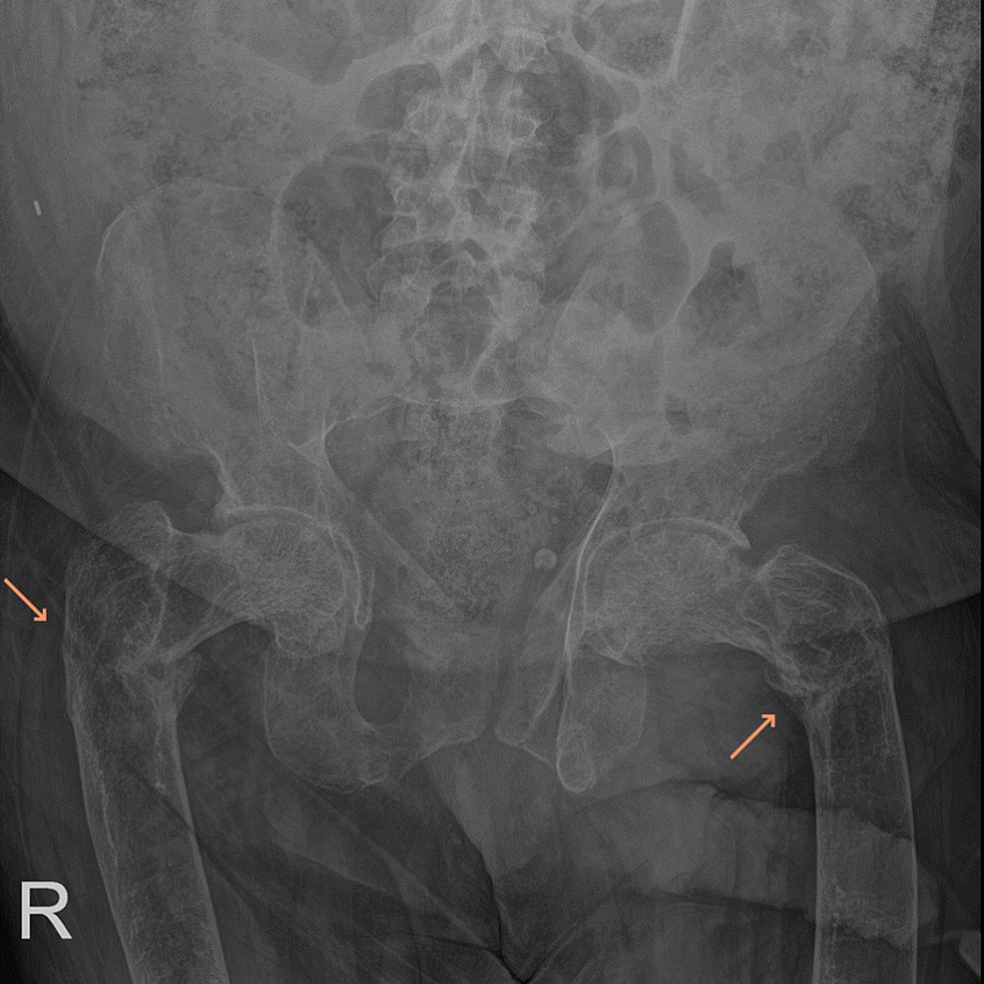
Pelvic radiograph showing generalized reduced bone density consistent with metabolic disease with healed bilateral proximal femoral fractures in the varus position (arrows).

**Figure 5:**
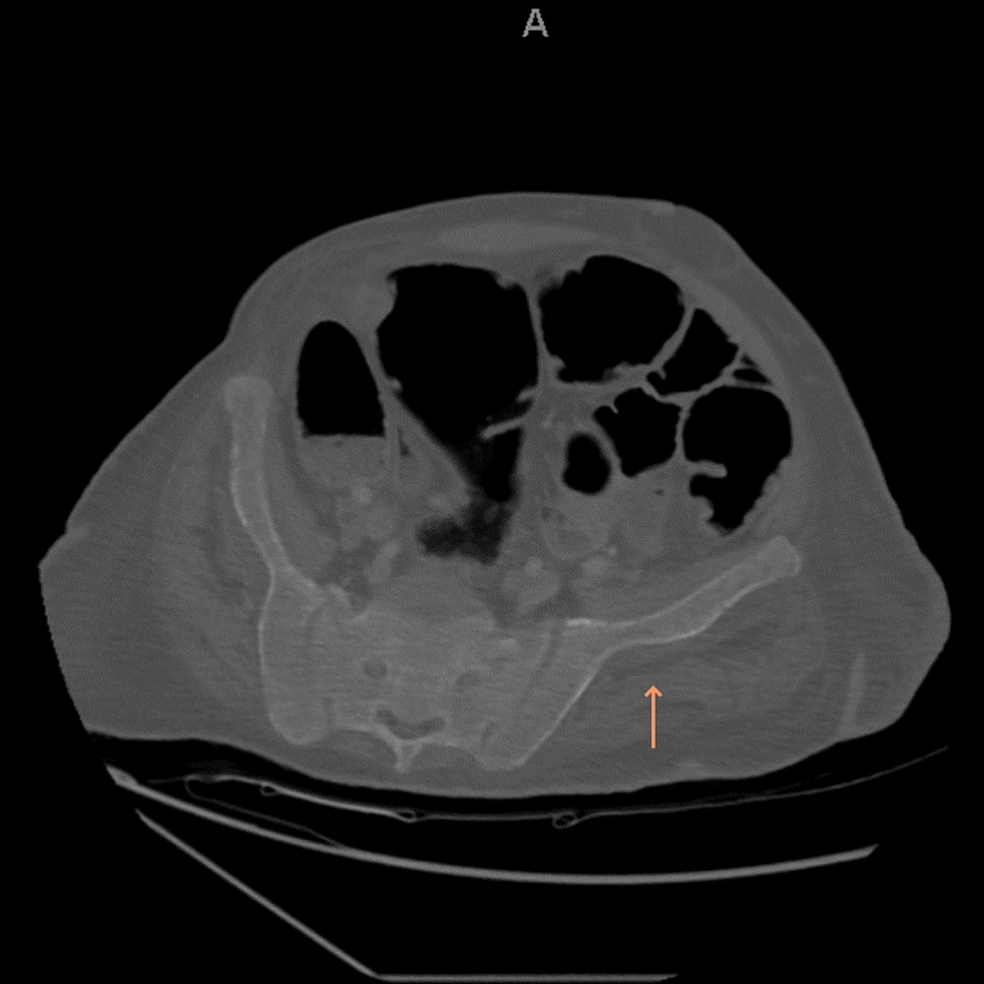
Pelvic computed tomography revealing a diffuse prominent trabeculation of the bone associated with osteopenia and minimal expansion, as well as multiple old fractures associated with pelvic deformity due to very soft bones (arrow). This is consistent with severe osteomalacia.

**Figure 6:**
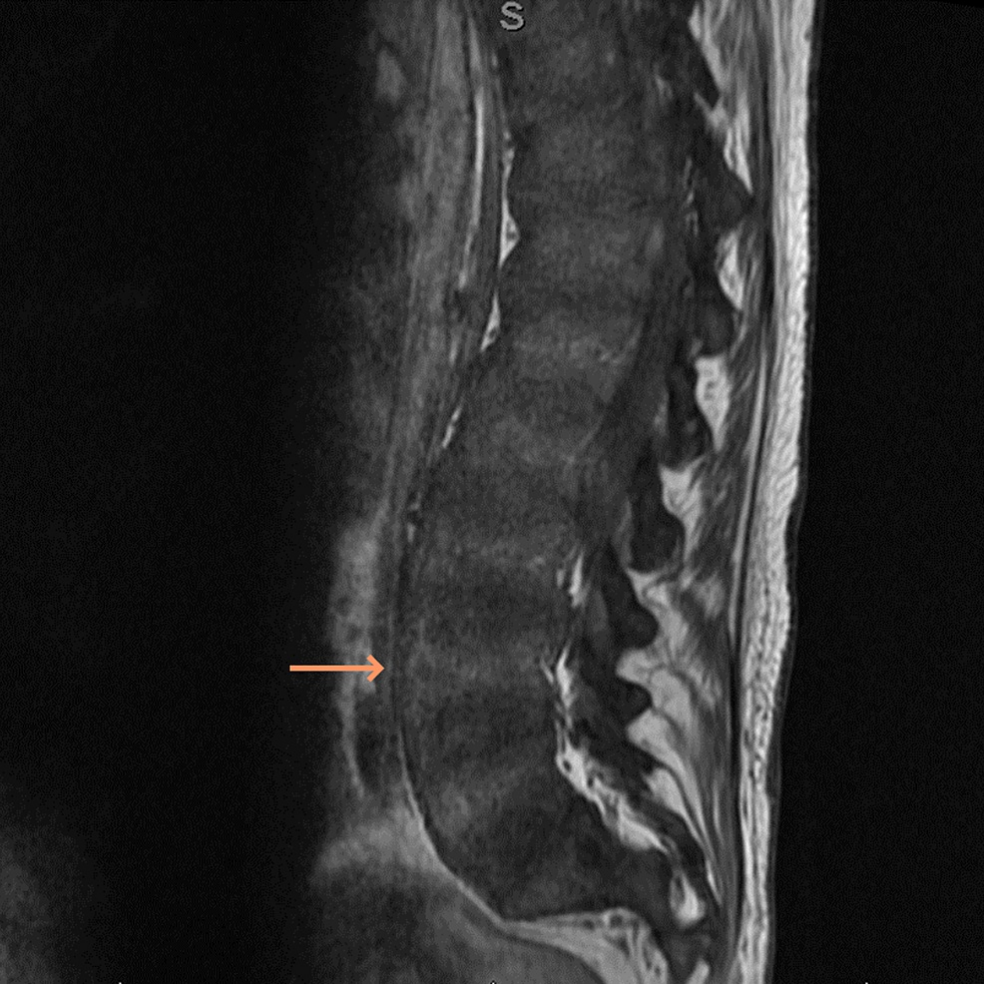
Lumbar spine magnetic resonance imaging revealing H-shaped vertebrae and fatty changes in the posterior paraspinal muscles (arrow), abnormal bone marrow signal intensity of the spine associated with metabolic disease.

On imaging, left and right shoulder radiographs revealed reduced bone density and old healed fractures as shown in Figure 1 and Figure 2. Chest radiography showed multiple old healed rib fractures as shown in Figure 3. Pelvic radiography showed reduced bone density and old healed proximal femoral fractures (Figure 4). Abdominal and pelvic computed tomography showed diffuse prominent trabeculation of the bones associated with osteopenia and minimal expansion, as well as multiple old fractures in the ribs bilaterally, spine, and pelvis (Figure 5). Furthermore, it is associated with deformity of the pelvic bones and rib cage most likely due to severe osteomalacia. Spine magnetic resonance imaging showed abnormal bone marrow signal intensity of the spine, ribs, and pelvic bone associated with metabolic bone disease with multiple bilateral rib fractures, cervical kyphosis, and thoracolumbar scoliosis (Figure 6).

In addition, venous blood gas (VBG) tests and echocardiography were conducted. Echocardiography revealed normal findings. However, the VBG test results showed respiratory acidosis and CO2 retention, for which a final diagnosis of respiratory failure type 1 complicating obstructive sleep apnea was established. BiPAP was given daily at night. The patient was admitted for 10 months to correct vitamin D and calcium levels. He was treated with oral vitamin D3 50,000 IU once daily, oral calcium carbonate 1200 mg three times per day, oral magnesium oxide 400 mg twice daily, oral one-alpha 4 µg once daily, oral ferrous sulfate 200 g once daily, potassium phosphate 0.25 mmol/kg IV infusion, and one tablet of oral Fefol daily. In addition, orthopedics was involved for fracture management, and he was started on physiotherapy. The main goal of management was to regain ambulation. After 10 months of treatment of vitamin D deficiency and correction of electrolyte imbalance, the patient was able to start standing on his legs, move with walking aids, rollover to each side of his bed, feed himself, and open a can, with improvement in memory function. In addition, his laboratory results improved as shown in Table 1.

## Discussion

Metabolic bone diseases after bariatric surgeries

Bone loss or decreased bone mineral density (BMD) has been reported in several studies after different types of bariatric surgeries [16]. There are currently three types of bariatric surgeries: (1) restrictive, in which the stomach size is reduced, such as laparoscopic adjustable gastric banding (LAGB); (2) malabsorptive, in which less food and nutrients are reabsorbed through the diversion of bile acids and pancreatic enzyme and shortening of the length of the bowel, such as biliopancreatic diversion with duodenal switch (BPD/DS); or (3) a combination of both, such as RYGB and SG [17]. Most of the studies reported consistent bone loss or decrease in BMD after RYGB particularly [16]. Several studies on this subject have focused on the decrease in BMD [16]. However, the extent of clinical metabolic bone diseases after bariatric surgeries has yet to be determined [18]. Osteopenia, osteoporosis, osteomalacia, osteomalacia-induced myopathy, and other complications of decreased bone density, such as fragility fractures, have been studied in a few studies or case reports only. In addition, there is an increase in the fracture risk after bariatric surgeries, which is found to be more with malabsorptive surgery than restrictive surgery [19].

Pathophysiology

There are several mechanisms by which bone loss after bariatric surgeries can be explained. The first mechanism is mechanical unloading, which is secondary to weight loss after bariatric surgeries [16,20]. Accordingly, less weight placed on the skeleton leads to less bone formation and more bone resorption, which leads to less BMD [16,20]. The second mechanism is calcium and vitamin D intestinal malabsorption with secondary hyperparathyroidism [16,20,21]. Given the alterations in the gastrointestinal tract anatomy with bariatric surgeries, subsequent vitamin D and calcium deficiencies are common [16,20,21]. Whenever serum calcium levels are low, parathyroid hormone (PTH) will be secreted, which subsequently causes bone resorption and loss of bone mass to increase serum calcium level [16]. The negative feedback to PTH is vitamin D (1,25-dihydroxyvitamin D (1,25-(OH)2D)) [16]. However, due to calcium and vitamin D deficiencies, continued release of PTH occurs, causing more bone resorption and lower BMD [16,20,21]. The third mechanism is hormonal changes after bariatric surgeries. Notably, loss of adipose tissue causes subsequent changes in the adipocyte hormonal balance. Adipocyte hormones, such as leptin and adiponectin, play an important role in bone mass regulation [16]. Leptin increases bone formation, whereas adiponectin exerts a negative impact on bone health [16,20,21]. After RYGB and SG, the loss of fat mass resulted in a decrease in serum leptin and an increase in adiponectin [16,20,21]. In addition, gut hormone changes after bariatric surgeries may impact bone metabolism [16,20,21].

To the best of our knowledge, only a few similar cases have been reported. In six similar cases (Table 2), malabsorptive surgery was done, two of whom underwent biliopancreatic diversion [16,22] and four underwent RYGB [23-26]. Compared with these cases, our case had worse outcomes in different domains. The first was related to the time of recognition and severity of presentation. Notably, other cases were diagnosed earlier, with a less severe presentation, such as insufficiency fractures, either femur fracture [24] or vertebral compression fractures [16,25], or presented with proximal muscle weakness [22,23,26]. However, our patient presented 10 years later with combined presentations. He had multiple fragility fractures in small and large bones, with myopathy and atrophy, which made him completely bedridden for more than five years. Second, our case had worse biochemical markers related to the presentation. Compared with all cases, our patient has the lowest laboratory findings, lowest vitamin D3 levels, lowest serum calcium levels, and lowest phosphorus levels. In addition, this case is rare and demonstrates what complications could develop when no vitamin and mineral supplementation is provided at all for up to nine years after SG and bypass surgeries.

**Table 2:** Comparison of our case with other similar case reports. *The original units in this table have been converted to SI units through the following conversion factors [27,28]: Vitamin D3: nmol/L = ng/mL × 2.496 Serum calcium: mmol/L = mg/dL × 0.25 Serum phosphorus: mmol/L = mg/dL × 0.323 Parathyroid hormone (PTH): ng/L = pg/mL × 1 ng/L = pmol/L × 9.5

Area of comparison	Our case report	Patient 1 ^[22]^	Patient 2 ^[23]^	Patient 3 ^[24]^	Patient 4 ^[25]^	Patient 5 ^[26]^	Patient 6 ^[16]^
Age	39	55	42	41	40	42	41
Gender	Male	Female	Female	Female	Female	Male	Male
Body mass index (before)	53.1	56	-	-	-	-	-
Body mass index (after)	-	31	-	45.8	-	-	-
Type of bariatric surgery	Biliopancreatic diversion	Biliopancreatic diversion	Roux-en-Y gastric bypass	Roux-en-Y gastric bypass	Roux-en-Y gastric bypass	Roux-en-Y gastric bypass	Biliopancreatic diversion
Supplements	No, for nine years	-	Yes	No, for eight years	Yes	Yes	-
Time of presentation after surgery	10 years	12 months	6 and a half years	8–10 years	5 months	6 months	2 years
Presentation	Bedridden with multiple fragility fractures, myopathy, and muscle atrophy	Proximal muscle weakness	Proximal muscle weakness	Insufficiency fracture (femur)	Multiple vertebral fractures	Proximal muscle weakness	Multiple vertebral compression fractures and rib fractures
Laboratory findings							
Vitamin D3*	0.1 ng/mL (= 0.25 nmol/L)	-	6 ng/mL (= 14.98 nmol/L)	7.0 ng/mL (= 17.47 nmol/L)	-	-	-
Serum calcium*	0.92 mmol/L	8.4 mg/dL (= 2.1 mmol/L)	8.6 mg/dL (= 2.15 mmol/L)	6.7 mg/dL (= 1.67 mmol/L)	2.53 mmol/L	-	6.18 mg/dL (= 1.54 mmol/L)
Serum phosphorus*	0.56 mmol/L	2.2 mg/dL (= 0.71 mmol/L)	3.3 mg/dL (= 1.065 mmol/L)	3.0 mg/dL (= 0.96 mmol/L)	1.06 mmol/L	-	2.11 mg/dL (= 0.68 mmol/L)
Parathyroid hormone (PTH)*	59.5 pmol/L (= 565.25 ng/L)	182.2 pg/mL (= 182.2 ng/L)	40 pmol/L (= 380 ng/L)	696.3 pg/mL (= 696.3 ng/L)	-	-	151.4 ng/L
Alkaline phosphatase (ALP)*	1017 U/L	456 U/L	653 U/L	164 U/L	-	-	-

Screening, supplementation, and exercise

Some cases had reported bone-related complications, such as fractures, shortly after surgery [25,26], which indicates the need for bone health screening before and after bariatric surgeries. Preoperatively, the patient should be counseled for bone loss risk after surgery and the importance of supplements [29-31]. In addition, screening of risk factors of bone loss, screening and correction of nutritional deficiencies including vitamin D deficiency, and dual-energy X-ray absorptiometry (DXA) scan to establish a baseline are all recommended preoperatively [29-31]. Postoperatively, routine annual screening of 25(OH)D serum levels is recommended with further modification on supplementation according to the patient’s need to replete any deficiencies [30,31]. DXA is recommended in the first two years after surgery for postmenopausal women and other adults with osteoporosis risk factors [30,31]. Serum calcium, PTH levels, and bone metabolic turnover markers may also be considered as postoperative follow-up screening [30,31]. The recent guidelines on postoperative vitamin D deficiency repletion recommend vitamin D3 (3000-6000 IU/day), which is a more potent treatment for repletion than D2, or vitamin D2 (50,000 IU one to three times weekly) [32]. Similarly, repletion of calcium deficiency is recommended and depends on the type of surgery. In particular, those who underwent LAGB, SG, and RYGB with calcium deficiency should take 1200-1500 mg/day, whereas those who underwent BPD/DS with calcium deficiency should take 1800-2400 mg/day [32]. Along with supplementation, regular weight-bearing exercise and aerobic exercise should be suggested and included in postoperative care [20,31]. According to a study evaluating the effects of exercise programs on obese patients who had undergone gastric bypass surgery, the supervised exercise program attenuated lumbar spine and right hip BMD loss while also improving lean mass and overall muscular strength, although it did not affect bone remodeling [33].

## Conclusions

The current patient had become bedridden due to severe vitamin D deficiency and hypocalcemia post-bariatric surgery. After 10 months of treatment, the patient had improved clinically such that he was able to stand unassisted. In addition, his serum vitamin D levels improved from 0.1 to 28 ng/mL, whereas his serum calcium levels improved from 0.92 to 2 mmol/L. Such a case emphasizes the urgent need for further research on the complications of late post-bariatric surgeries. Moreover, our findings support the need for increased awareness among both patients and physicians on anti-obesity medication as a noninvasive alternative for bariatric surgeries. Our take-home message is to increase awareness of patient supplementation compliance and incorporation of early detection and intervention to avoid disastrous consequences on the patient’s health. Patients scheduled to undergo surgical intervention for weight reduction must be aware of hypovitaminosis consequences.
